# Reevaluating hikikomori and challenging loneliness assumptions in Japan: A cross-sectional analysis of a nationwide internet sample

**DOI:** 10.3389/fpsyt.2024.1323846

**Published:** 2024-02-15

**Authors:** Roseline Yong

**Affiliations:** ^1^ Department of Mental Health, Graduate School of Medicine, The University of Tokyo, Tokyo, Japan; ^2^ Department of Environmental Health Science and Public Health, Graduate School of Medicine, Akita University, Akita, Japan

**Keywords:** loneliness, hikikomori, social withdrawal, outgoing behaviors, mental health, cross-sectional, secondary analysis, nationwide sample

## Abstract

**Introduction:**

Loneliness in Japan, accentuated by demographic challenges and the hikikomori phenomenon (extreme social withdrawal), has raised concerns. This study critically examines loneliness dynamics, questioning assumptions embedded in hikikomori classifications. The term “hikikomori,” originally signifying prolonged home stay, requires nuanced exploration, especially regarding outgoing behaviors’ relationship with loneliness.

**Objectives:**

Investigating the intricate connection between outgoing behaviors and loneliness, this study questions the effectiveness of existing hikikomori classifications. Aiming to understand if these classifications accurately represent the loneliness spectrum across age groups, the research emphasizes the significance of comprehending loneliness dynamics amid societal challenges. The study explores an array of factors influencing loneliness, including demographics, mental health, and outgoing behaviors, advocating for a reassessment of assumptions linked to hikikomori classifications.

**Methods:**

This secondary analysis employed data from a nationwide Internet addiction survey conducted in July 2012. A sample of 623 participants, representative of Japanese internet users aged 16 and above, was included for analysis. Loneliness was assessed using the UCLA Loneliness Scale, and exposure variables included demographic, mental health, outgoing behaviors, and lifestyle factors. Statistical analyses encompassed descriptive statistics, one-way ANOVA, chi-square tests, and logistic regression.

**Results:**

Significant differences were observed in loneliness scores based on sex, age, marital status, employment, and outgoing behaviors. Mental health factors, including dissatisfaction with life and romance, life stress, and psychological distress, emerged as strong contributors to loneliness. The study challenges existing hikikomori classifications, suggesting they may not fully encapsulate the loneliness experiences of individuals engaged in routine school or work activities.

**Conclusion:**

Findings underscore the need for a reevaluation of hikikomori, emphasizing loneliness as a complex and multifaceted issue in Japan. The study advocates for nuanced strategies to address loneliness, considering diverse demographic vulnerabilities. Limitations include the pre-pandemic sample and potential unmeasured confounding factors.

## Introduction

The subject of loneliness has gained recognition and garnered particular attention, exacerbated by an amalgamation of demographic challenges and the emergence of the social phenomenon known as hikikomori. This study delves into the intricate dynamics of loneliness, both during the pre-pandemic era and in response to the amplified loneliness during the COVID-19 pandemic.

Hikikomori, a term associated with extreme social withdrawal, is often intertwined with loneliness and isolation. Initially perceived as a uniquely Japanese phenomenon, hikikomori was defined as staying at home and refraining from social activities for over six months, without a preliminary psychosis background (1). Over time, the “hikikomori support guidelines” expanded the definition to include individuals who may go out but lack meaningful social interactions (2, 3). The duration defining hikikomori has also been variably considered, ranging from 3-6 months in some studies (4–7).

Outgoing patterns have traditionally served as a means to differentiate hikikomori individuals from those who are not. These patterns have given rise to two distinct categories: the non-hikikomori group, including individuals who maintain regular work or school attendance or engage in various social activities, and the hikikomori group, comprising individuals who predominantly remain at home, with limited outings for personal interests or nearby errands (8–10). However, beneath this classification lies a fundamental question: Does the characterization of outgoing behaviors effectively encapsulate the diverse experiences of loneliness among hikikomori individuals? This pivotal query forms the basis for our exploration of the intricate relationship between outgoing behaviors and loneliness.

For example, in previous studies (8–10) on hikikomori prevalence, participants selected one of eight outgoing patterns:

1. Going out for work or school every day.
2. Going out for work and school 3-4 days a week.
3. Going out for fun frequently.
4. Sometimes going out to maintain relationships with others.
5. Mainly staying home yet sometimes going out for tasks concerning self-interest.
6. Mainly staying home yet could go out to neighboring convenient shops.
7. Can come out from the room but cannot go out from the house.
8. Almost never going out from the room.

Participants who selected options 5-8 were classified as hikikomori. Analyzing these criteria, option no. 4 might resemble an individual who primarily stays home but attends funerals and significant family events to show respect and maintain minimal social interactions with others. As for option no. 5, it may resemble the phenomenon of a freelancer or home-based Otaku (meaning: a person obsessed with computers or particular aspects of popular culture to the detriment of their social skills) (11). Those previously classified as non-hikikomori for choosing options 1-4 might not accurately be non-hikikomori. Leveraging the broadened definition of hikikomori in 2010 (3), those who choose option 2 may reflect a socially withdrawn person attending school or work for a limited time each week. People who choose option no. 4 can almost be identified as hikikomori according to the definition, and option no. 5 may fulfill the definition of hikikomori, yet there is a positive motivation for the condition. These criteria have not rightly reflected the motivation or interest of a person, thus failing to represent hikikomori accurately. Therefore, I question the rationale and utility of these criteria in classifying hikikomori characters. This crucial inquiry forms the basis for this exploration of the intricate relationship between outgoing behaviors and loneliness.

Concerns about hikikomori span across all ages, necessitating a critical assessment to determine if its classification genuinely reflects the full spectrum of loneliness experiences in Japan. It is also crucial to ascertain whether the degree of loneliness is consistent across all age groups.

Amid the pandemic, the profound implications of loneliness for mental health have come to the forefront, intensifying research and raising significant concerns (12) The impact of loneliness has been especially pronounced in Japan, where comprehensive online surveys have unveiled a surge in loneliness, with notable disparities between women and men (12). Recognizing the severity of harmful effects on mental and physical well-being due to loneliness or social isolation, a new law (to be implemented on 2024.4.1) has been enacted to establish principles, state responsibilities, and policy matters for loneliness and isolation measures, Act No.45 of 2023 (13). These measures aim to prevent loneliness and isolation, provide prompt support, and promote efforts to break free from these states.

However, the critical distinction between pre-pandemic and pandemic-induced loneliness emerges as a central theme of inquiry. While the latter may wane as pandemic-related restrictions ease, the former may endure, warranting a comprehensive understanding of its prevalence and implications.

Previous research has associated loneliness with diverse factors such as unemployment (14) and hikikomori (15, 16), the role of outgoing patterns in this context remains a relatively uncharted territory. Similarly, the connections between life satisfaction, marital status, age, and loneliness have demonstrated multifaceted and at times, contrasting relationships (17–21). These complex interactions underscore the need to revisit and reconsider long-held assumptions surrounding these factors when examining loneliness in Japan.

In light of these considerations, this study embarks on a comprehensive investigation into the contributors to loneliness in Japan. It takes into account a spectrum of demographic factors, including age, marital status, education, occupational status, and outgoing patterns. This study aims to investigate loneliness by examining various outgoing behaviors, questioning the usefulness of outgoing patterns in classifying hikikomori, and highlighting the need for a more nuanced approach. Additionally, this research critically examines the classification of hikikomori as an indicator of social isolation and loneliness, aiming to disentangle the intricate web of loneliness in Japanese society. The findings challenge preconceived notions about the hikikomori label as a comprehensive representation of loneliness experiences in Japan. As Japan grapples with the pressing issue of loneliness, this study calls for a reexamination of hikikomori, challenging assumptions, and redefining loneliness within the complex dimensions of the country.

## Methods

### Sample recruitment

In this study, I conducted a secondary analysis of data derived from a July 2012 Internet addiction population survey (22). The original survey aimed to create a representative sample by mirroring the national population of Internet users aged 16 and above in 2010 (23). Participants were recruited through Macromill, an Internet survey company with a database of 1,086,904 registered users in May 2012. The registered users were stratified by sex and age, assigned pseudorandomized numbers, and sorted accordingly, resulting in a randomized data list.

Participants were initially categorized by sex and age groups and then randomly chosen from the user database. Invitations to participate in the survey were sent to 4,886 registered users via email, resulting in 623 participants (with a successful response rate of 12.7%) after data cleaning. Prior to taking the survey, participants were required to provide consent. Access to the online survey followed a ‘first come, first served’ basis, disabling the survey link once the quota for an effective sample size for the gender and age range had been met. Considering the total time for survey completion, invitation emails were sent at 9 am on Saturday, July 28, 2012, aiming to accommodate working individuals and students. The sampling quota was estimated to be fulfilled by the following Monday, approximately within 48–52 hours. Ethical approval for the initial study was obtained from the Research Ethics Committee of the Graduate School of Medicine at the University of Tokyo.

Outcome variable. Loneliness was assessed using a 20-item 4-Likert scale (UCLA Loneliness Scale) (α = 0.87–0.91) (24).

Exposure variables. These variables were considered to understand the complex interplay of factors contributing to loneliness in the study population, allowing for a comprehensive analysis of the issue.

Demographic variables: sex (men, women), age categories (16-19, 20-29, 30-39, 40-49, 50-59, 60 and above), educational level (compulsory education, high school/vocational school education, university education), single, preferred not to tell), employment variables (working, housework, studying, not working), marital status (married/cohabitated/dating, widowed/divorced/separated.

Mental health variables: satisfactions (romance satisfaction in the current romance stats, life satisfaction in the current job/school/situation), life stress in the current job/school/situation, psychological distress (6-item 5-Likert scale (K6) (α = 0.85, 0-8=no/mild, 9-24=moderate/severe) (25).

### Outgoing behaviors variables

Building upon the original set of outgoing patterns used to distinguish hikikomori individuals from those who are not (8–10), this study introduces a novel array of outgoing behavior variables. These variables encompass diverse levels of social engagement and outdoor activities, spanning individuals who are frequently active outside to those who predominantly prefer indoor settings. The new categories include individuals who:

1. Go out for work or school and frequently engage in other activities outside the home.
2. Go out for work or school and attend to other errands as needed.
3. Go out for work or school but refrain from going out for other reasons.
4. Do not go to work or school but frequently engage in outings for leisure or other activities.
5. Mostly stay at home but occasionally go out for social events like ceremonies or weddings.
6. Usually stay at home but go out only when the activity is related to personal interest.
7. Typically stay at home but venture out to nearby places like convenience stores or rarely leave their room/home.

Participants were asked to select one criterion that best represented their outgoing pattern in the past six months. Those who chose option 7 were classified as hikikomori.

Lifestyle variables: various everyday Internet use habits (e.g., study/work, stress release, killing time, communication, associating, expanding hobbies and network of friends, resourcing, sharing problems, building community networks, online dating, assessing pornography, using anonymous online bulletin boards, blogging and SNS, releasing personal updates and work presentations, assessing Youtube or iTunes, using P2P and FTP, online gaming, online survey or quiz, financial transaction, and online shopping or auctions).

### Statistical analysis

The statistical analysis commenced with an examination of descriptive statistics, which encompassed measures such as the mean, median, mode, and the assessment of normality for the UCLA Loneliness Score. These scores were subsequently categorized into “lower scores” and “higher scores,” with a specific focus on the latter as the dependent variable of interest. Following this categorization, we performed a one-way ANOVA to facilitate the comparison of means. Chi-square test were used to explore the relationship between loneliness and its exposure variables. To ensure the validity of our statistical tests, we assessed the homogeneity of variances using Levene’s and Welch’s tests. Additionally, we calculated effect sizes, employing Cohen’s classification for eta-squared to gauge the magnitude of effects. Specifically, we categorized effects as small (.01), medium (.06), or large (.14). Furthermore, we explored the strength and direction of linear relationships between variables through the Pearson correlation test. Concurrently, logistic regression analysis was carried out, taking into consideration two distinct models. Model 1 incorporated all relevant demographic variables (items that had statistically significant influence on loneliness in chi-square test for independence) as potential confounding variables, and Model 2 included all relevant internet use habits and all relevant demographic variables as potential confounding factors. This comprehensive statistical analysis provides valuable insights into the factors influencing higher UCLA loneliness scores within our diverse sample.

## Results

UCLA Loneliness scores exhibited a range of 20 to 80 within the sample, with a mean score of 42.4 (SD=12.1, 5% trimmed mean=41.9), a median score of 41.0, and a mode of 40. Scores between 20 and 40 were classified as “lower scores,” while scores between 41 and 80 were designated “higher scores.” Correlations among most variables were characterized by small effect sizes (r<.30), with exceptions observed in the relationships between marital status and age (r=.343, p<.001), as well as between romance satisfaction (r=.330, p<.001).

Demographic variables. Significant differences were observed in the UCLA loneliness scores among various factors (**Table 1**). Sex: Men had higher UCLA loneliness scores, with a mean of 44.4 (SD = 12.5), while women had a lower mean score of 40.2 (SD = 11.4). The effect size (eta squared) was small at 0.03, indicating a small but significant difference in loneliness scores between the sexes. Age: Age groups exhibited significant differences in loneliness scores (p < 0.001). Notably, individuals in their 10s and 60s showed higher loneliness scores than other age groups. For example, those in their 10s had a mean loneliness score of 47.1 (SD = 14.3), while individuals in their 60s had a mean score of 39.5 (SD = 10.6). Employment status: Employment status significantly differed in UCLA loneliness scores (p < 0.001). Individuals not working had the highest loneliness scores, with a mean of 47.9 (SD = 13.9), while those working had the lowest mean score of 42.0 (SD = 11.8). The effect size was moderate at 0.04. Marital status: A significant difference was observed among different marital status categories (p < 0.001). Participants who were single had the highest UCLA loneliness scores, with a mean of 48.9 (SD = 14.3). In contrast, those who were married, cohabited, or dating had the lowest mean score of 39.6 (SD = 10.2). The effect size was relatively large at 0.09, indicating a substantial difference in loneliness scores based on marital status.

**Table 1:** Distributions of UCLA loneliness scores with different demographics, mental health, and outgoing behaviors subgroups.

		UCLA loneliness scores			Mean	SD	Eta squared
		lower scores	higher scores	*p* value			
**Sex**	men	213 (47.1%)	110 (64.3%)	<.001	44.4	12.5	0.03
	women	239 (52.9%)	61 (35.7%)		40.2	11.4	
**Areas**	Hokkaido	18 (4%)	10 (5.8%)	0.656	46.1	12.9	0.01
	Tohoku	18 (4%)	7 (4.1%)		42.0	11.8	
	Kanto	177 (39.2%)	66 (38.6%)		42.3	12.3	
	Chubu	59 (13.1%)	31 (18.1%)		44.0	13.1	
	Kinki	109 (24.1%)	36 (21.1%)		41.5	11.7	
	Chukoku	20 (4.4%)	7 (4.1%)		43.1	11.5	
	Shikoku	15 (3.3%)	5 (2.9%)		40.3	10.7	
	Kyushu	36 (8%)	9 (5.3%)		40.7	11.6	
**Age**	10's	23 (5.1%)	21 (12.3%)	<.001	47.1	14.3	0.03
	20's	68 (15%)	32 (18.7%)		44.5	13.8	
	30's	93 (20.6%)	37 (21.6%)		42.2	12.2	
	40's	87 (19.2%)	37 (21.6%)		42.2	11.5	
	50's	73 (16.2%)	27 (15.8%)		42.1	11.3	
	60's	108 (23.9%)	17 (9.9%)		39.5	10.6	
Educational level	Compulsory education	6 (1.3%)	2 (1.2%)	0.844	44.0	14.6	0.01
	High school/ technical school education	192 (42.5%)	77 (45.0%)		43.4	11.8	
	Univeresity level education	254 (56.2%)	92 (53.8%)		41.5	12.3	
Employment	Working	263 (58.2%)	97 (56.7%)	<.001	42.0	11.8	0.04
	Housework	102 (22.6%)	19 (11.1%)		39.2	9.8	
	Studying	39 (8.6%)	20 (11.7%)		43.5	13.4	
	Not working	48 (10.6%)	35 (20.5%)		47.9	13.9	
Marital status	Married/cohabited/dating	321 (71%)	72 (42.1%)	<.001	39.6	10.2	0.09
	Widowed/divorced/separated	41 (9.1%)	22 (12.9%)		43.9	13.2	
	Single	64 (14.2%)	60 (35.1%)		48.9	14.3	
	Preferred not tell	26 (5.8%)	17 (9.9%)		46.3	11.8	
Romance satisfaction	Satisfied	304 (67.3%)	81 (47.4%)	<.001	40.1	11.4	0.05
	Unsatisfied	26 (5.8%)	17 (9.9%)		46.0	12.6	
	Preferred not to tell	122 (27.0%)	73 (42.7%)		46.3	11.8	
Life satisfaction	Satisfied	273 (60.4%)	42 (24.6%)	<.001	37.8	10.1	0.15
	Unsatisfied	179 (39.6%)	129 (75.4%)		47.1	12.3	
Life stress	Not stress	226 (50%)	52 (30.4%)	<.001	38.8	10.9	0.07
	Stress	226 (50%)	119 (69.6%)		45.2	12.3	
Psychological distress	K6 scores less than 9	408 (90.3%)	104 (60.8%)	<.001	39.9	10.6	0.19
	K6 scores >9	44 (9.7%)	67 (39.2%)		53.6	12.4	
Outgoing behaviors	goes out for work or school and frequently goes out for other reasons as well	113 (25%)	28 (16.4%)	0.028	38.6	11.3	0.05
	goes out for work or school and goes out when there are other errands to attend to	186 (41.2%)	65 (38%)		42.1	11.9	
	goes out for work or school but doesn't go out for other reasons	7 (1.5%)	9 (5.3%)		52.1	12.0	
	doesn't go to work or school but frequently goes out for leisure or other activities	21 (4.6%)	8 (4.7%)		41.3	10.6	
	mostly stays at home but occasionally goes out for social events like ceremonies or weddings	29 (6.4%)	15 (8.8%)		45.0	12.5	
	usually stays at home but goes out only when it's related to personal interest	79 (17.5%)	36 (21.1%)		44.4	12.0	
	typically stays at home but goes to nearby places like convenience stores or rarely leave room/home	17 (3.8%)	10 (5.8%)		47.0	14.0	

Chi-square test were used to explore the relationship between loneliness and its exposure variables.  To ensure the validity of our statistical tests, the homogeneity of variances using Levene's and Welch's tests were assessed.  One-way ANOVA to facilitate the comparison of means. Effect sizes, employing Cohen's classification for eta-squared were calculated to gauge the magnitude of effects, small (.01), medium (.06), or large (.14). (N=623).

Mental health variables. Marital status satisfaction: The level of satisfaction with one’s marital status also yielded a significant difference in loneliness scores (p < 0.001). Participants who were unsatisfied or preferred not to reveal their satisfaction had higher UCLA loneliness scores, with a mean of 46.0 (SD = 12.4), compared to those who were satisfied, with a mean score of 40.1 (SD = 11.4). The effect size was moderate at 0.05. Life satisfaction: The satisfaction with one’s current situation significantly impacted loneliness scores (p < 0.001). Participants who were unsatisfied with their current situation had higher UCLA loneliness scores, with a mean of 47.1 (SD = 12.3), compared to those who were satisfied, with a mean score of 37.8 (SD = 10.1). The effect size was relatively large at 0.15. Life stress: The presence of stress in the current situation significantly affected loneliness scores (p < 0.001). Those who reported stress had higher UCLA loneliness scores, with a mean of 45.2 (SD = 12.3), compared to individuals without stress, who had a mean score of 38.8 (SD = 10.9). The effect size was moderate at 0.07. **K6 scores:** The psychological distress measured by K6 scores showed a significant difference (p < 0.001). Individuals with higher distress (K6 scores >9) had substantially higher UCLA loneliness scores, with a mean of 53.6 (SD = 12.4), compared to those with lower distress (K6 scores 0-8), who had a mean score of 39.9 (SD = 10.6). The effect size was large at 0.19.

Outgoing behaviors. Different patterns of outgoing behavior also demonstrated significant differences in loneliness scores (p = 0.028). Participants who attended school/work and were outgoing had the lowest mean loneliness score (38.6, SD = 11.3), while individuals who only attended school/work but did not go out had the highest mean score (52.1, SD = 12.0). The effect size was close to moderate at 0.05.

Internet use habits. Significant differences in UCLA loneliness scores were observed based on varying online activities (**Table 2**). Notably, the frequency of using the internet for stress release and killing time showed significant associations with loneliness. Participants who used the internet to release stress (mean=44.4, SD=13.6) or kill time (mean=44.0, SD=13.2) “often/always” reported higher levels of loneliness compared to those who did so “rarely/never.” A similar pattern emerged for online dating, assessing pornography, using anonymous online bulletin boards, and accessing platforms like Youtube/iTunes. In these cases, frequent engagement with these online activities was associated with increased loneliness scores. However, it’s important to note that the effect sizes for these associations were relatively small, indicating that while statistically significant, the practical significance may be limited.

**Table 2:** Distributions of UCLA loneliness scores among different Internet use habits.

		UCLA loneliness scores			Mean	SD	Eta squared
		lower scores	higher scores	*p* value			
Official purpose (study/work)	rarely/never	211 (46.7%)	72 (42.1%)	0.238	43.0	12.1	0.00
	sometimes	81 (17.9%)	26 (15.2%)		41.9	12.0	
	often/always	160 (35.4%)	73 (42.7%)		41.8	12.2	
Stress release	rarely/never	201 (44.5%)	60 (35.1%)	0.001	40.9	12.0	0.02
	sometimes	128 (28.3%)	38 (22.2%)		42.2	10.2	
	often/always	123 (27.2%)	73 (42.7%)		44.4	13.6	
Killing time	rarely/never	105 (23.2%)	25 (14.6%)	0.005	38.9	10.6	0.03
	sometimes	123 (27.2%)	37 (21.6%)		41.8	10.3	
	often/always	224 (49.6%)	109 (63.7%)		44.0	13.2	
Communication	rarely/never	223 (49.3%)	96 (56.1%)	0.267	43.7	12.3	0.02
	sometimes	116 (25.7%)	41 (24%)		42.0	11.7	
	often/always	113 (25%)	34 (19.9%)		39.8	11.8	
Associating	rarely/never	359 (78.8%)	128 (74.9%)	0.026	42.2	12.3	0.00
	sometimes	59 (13.1%)	17 (9.9%)		41.8	11.4	
	often/always	37 (8.2%)	26 (15.2%)		44.4	11.9	
Expanding hobbies and network of friends	rarely/never	341 (75.4%)	132 (77.2%)	0.549	42.6	12.2	0.00
	sometimes	56 (12.4%)	16 (9.4%)		41.3	10.0	
	often/always	55 (12.2%)	23 (13.5%)		41.8	13.7	
Resourcing	rarely/never	26 (5.8%)	5 (2.9%)	0.258	42.1	8.7	0.00
	sometimes	59 (13.1%)	19 (11.1%)		42.6	11.0	
	often/always	367 (81.2%)	147 (86%)		42.4	12.5	
Sharing problems	rarely/never	376 (83.2%)	135 (78.9%)	0.46	42.0	12.2	0.01
	sometimes	50 (11.1%)	23 (13.5%)		44.1	11.1	
	often/always	26 (5.8%)	13 (7.6%)		44.5	12.6	
Building community network	rarely/never	388 (85.8%)	153 (89.5%)	0.421	42.8	12.4	0.01
	sometimes	27 (6%)	9 (5.3%)		40.6	9.5	
	often/always	37 (8.2%)	9 (5.3%)		38.7	10.6	
Online dating	rarely/never	441 (97.6%)	158 (92.4%)	0.011	42.1	12.1	0.01
	sometimes	5 (1.1%)	5 (2.9%)		47.5	10.4	
	often/always	6 (1.3%)	8 (4.7%)		50.6	12.9	
Assessing pornography	rarely/never	371 (82.1%)	121 (70.8%)	0.008	41.5	11.9	0.02
	sometimes	49 (10.8%)	31 (18.1%)		45.4	12.5	
	often/always	32 (7.1%)	19 (11.1%)		45.8	13.0	
Using anonymous online bulletin boards (2ch etc.)	rarely/never	316 (69.9%)	93 (54.4%)	<.001	40.9	11.3	0.03
	sometimes	87 (19.2%)	40 (23.4%)		44.0	12.2	
	often/always	49 (10.8%)	38 (22.2%)		46.9	14.4	
Blogging and SNS	rarely/never	219 (48.5%)	83 (48.5%)	0.894	42.3	12.1	0.00
	sometimes	86 (19%)	35 (20.5%)		43.3	11.4	
	often/always	147 (32.5%)	53 (31%)		41.9	12.7	
Release personal updates and work presentations	rarely/never	380 (84.1%)	139 (81.3%)	0.246	42.4	12.2	0.00
	sometimes	32 (7.1%)	19 (11.1%)		44.2	11.7	
	often/always	40 (8.8%)	13 (7.6%)		40.6	11.8	
Assessing Youtube/iTunes etc.	rarely/never	210 (46.5%)	54 (31.6%)	0.003	40.8	11.0	0.01
	sometimes	114 (25.2%)	53 (31%)		44.1	12.3	
	often/always	128 (28.3%)	64 (37.4%)		43.1	13.3	
P2P and FTP	rarely/never	364 (80.5%)	128 (74.9%)	0.298	42.3	12.4	0.00
	sometimes	52 (11.5%)	25 (14.6%)		42.7	10.3	
	often/always	36 (8%)	18 (10.5%)		42.6	12.7	
Online gaming	rarely/never	382 (84.5%)	134 (78.4%)	0.05	41.9	12.2	0.01
	sometimes	43 (9.5%)	17 (9.9%)		43.5	10.7	
	often/always	27 (6%)	20 (11.7%)		46.0	12.8	
Online survey or quiz	rarely/never	27 (6%)	12 (7%)	0.891	46.0	12.6	0.01
	sometimes	78 (17.3%)	29 (17%)		42.2	11.7	
	often/always	347 (76.8%)	130 (76%)		42.1	12.2	
Financial transaction	rarely/never	227 (50.2%)	96 (56.1%)	0.294	43.2	12.5	0.01
	sometimes	110 (24.3%)	41 (24%)		42.9	11.6	
	often/always	115 (25.4%)	34 (19.9%)		40.0	11.7	
Online shopping/auctions	rarely/never	99 (21.9%)	33 (19.3%)	0.393	41.6	10.8	0.00
	sometimes	174 (38.5%)	60 (35.1%)		42.5	11.6	
	often/always	179 (39.6%)	78 (45.6%)		42.7	13.2	

Chi-square test were used to explore the relationship between loneliness and various everyday internet use habits.  To ensure the validity of our statistical tests, the homogeneity of variances using Levene's and Welch's tests were assessed.  One-way ANOVA to facilitate the comparison of means. Effect sizes, employing Cohen's classification for eta-squared were calculated to gauge the magnitude of effects, small (.01), medium (.06), or large (.14). (N=623).

The logistic regression results were presented in **Table 3**. In Model 1, sex, age, marital status and employment status were adjusted for confounding factors. Demographic variables. Men had significantly higher odds of experiencing the outcome compared to women (OR: 1.96, 95% CI: 1.26-3.06). Several age groups showed significant associations with the outcome. Participants in their 10s had substantially higher odds of experiencing the outcome (OR: 16.02, 95% CI: 4.51-56.90). Those in their 20s (OR: 4.05, 95% CI: 1.79-9.15), 30s (OR: 3.21, 95% CI: 1.53-6.75), 40s (OR: 3.62, 95% CI: 1.70-7.71), and 50s (OR: 3.51, 95% CI: 1.62-7.59) also had significantly higher odds compared to those aged 60 years and above. Individuals who were single had significantly higher odds of experiencing the outcome (OR: 3.34, 95% CI: 1.98-5.63) compared to those who were married, cohabited, or dating. Being employed (working) (OR: 0.36, 95% CI: 0.19-0.68). and students (OR: 0.10, 95%CI: 0.03-0.32) were associated with significantly lower odds of experiencing moderate high to severe loneliness compared to those who were not working. Individuals who were unsatisfied with their romance status had 2.65 times higher odds (95% CI: 1.29 to 5.46) of experiencing higher levels of loneliness compared to those who were satisfied with their romance. Participants who were unsatisfied with their job had 3.48 times higher odds (95% CI: 2.27 to 5.33) of experiencing higher levels of loneliness compared to those who were satisfied.

**Table 3:** Logistic Regression Analysis of Factors Influencing Loneliness.

		Model 1			Model 2		
		OR	95%CI		OR	95%CI	
Sex	men	1.96	1.26	3.06	1.82	1.11	3.00
	women	ref					
Age	10's	16.02	4.51	56.90	13.03	3.47	48.99
	20's	4.05	1.79	9.15	2.96	1.23	7.17
	30's	3.21	1.53	6.75	2.61	1.18	5.77
	40's	3.62	1.70	7.71	2.40	1.55	7.46
	50's	3.51	1.62	7.59	3.30	1.50	7.28
	60 years and above	ref			ref		
Educational level	compulsory education	ref			ref		
	high school/vocational school education	1.65	0.30	9.08	2.16	0.35	13.16
	university level education	1.62	0.30	8.91	2.07	0.34	12.69
Employment	working	0.36	0.19	0.68	0.36	0.19	0.69
	housework	0.50	0.22	1.16	0.54	0.229	1.25
	studying	0.10	0.03	0.32	0.10	0.03	0.33
	not working	ref			ref		
Marital status	married/cohabited/dating	ref			ref		
	widowed/divorced/separated	2.04	1.09	3.84	1.89	0.99	3.60
	single	3.34	1.98	5.63	2.94	1.71	5.03
	preferred not to tell	2.37	1.16	4.82	2.22	1.06	4.72
Romance satisfaction	Satisfied	ref			ref		
	Unsatisfied	2.65	1.29	5.46	2.50	1.17	5.35
	Preferred not to tell	1.50	0.97	2.32	1.51	0.97	2.36
Life satisfaction	Satisfied	ref			ref		
	Unsatisfied	3.48	2.27	5.33	3.50	2.26	5.41
Life stress	Not stress	ref			ref		
	Stress	1.95	1.28	2.96	1.96	1.27	3.02
Psychological distress	K6 scores >9	4.76	2.91	7.81	4.63	2.78	7.69
	K6 scores less than 9	ref			ref		
Outgoing behaviors	goes out for work or school and frequently goes out for other reasons as well	ref			ref		
	goes out for work or school and goes out when there are other errands to attend to	1.40	0.82	2.39	1.43	0.83	2.47
	goes out for work or school but doesn't go out for other reasons	3.96	1.25	12.51	4.13	1.27	13.39
	doesn't go to work or school but frequently goes out for leisure or other activities	1.81	0.61	5.34	1.82	0.59	5.61
	mostly stays at home but occasionally goes out for social events like ceremonies or weddings	2.93	1.16	7.43	3.19	1.23	8.28
	usually stays at home but goes out only when it's related to personal interest	1.80	0.64	5.03	2.27	1.12	4.61
	typically stays at home but goes to nearby places like convenience stores or rarely leave room/home	1.98	1.26	3.12	2.09	0.71	6.12

Model 1 incorporated all relevant demographic variables (items that had statistically significant influence on loneliness in chi-square test for independence) as potential confounding variables, and Model 2 included all relevant internet use habits and all relevant demographic variables as potential confounding factors. (N=623).

Life Stress: Participants who reported being stressed had significantly higher odds of experiencing the outcome (OR: 1.95, 95% CI: 1.28-2.96). K6 Scores: Participants with K6 scores greater than 9 had significantly higher odds of experiencing the outcome (OR: 4.76, 95% CI: 2.91-7.81) compared to those with scores less than 9. Outgoing behaviors: Participants who reported “going out for work or school but doesn’t go out for other reasons” (OR: 3.96, 95% CI: 1.25-12.51), “mostly stays at home but occasionally goes out for social events like ceremonies or weddings” (OR: 2.93, 95% CI: 1.16-7.43), “typically stays at home but goes to nearby places like convenience stores or rarely leave room/home” (OR: 1.98, 95% CI: 1.26-3.12),had significantly higher odds of experiencing the outcome compared to those who “goes out for work or school and frequently goes out for other reasons as well.” The effect of satisfaction in life and romance on loneliness were not statistically significant.

The inclusion of Internet use patterns in Model 2 did not substantially change the interpretation of results for most predictor variables when compared to Model 1. However, participants who “usually stay at home but goes out only when it’s related to personal interest” had significantly higher odds of experiencing the outcome (OR: 2.27, 95% CI: 1.12 to 4.61) compared to the reference group, while the impact of participants who “typically stays at home but goes to nearby places like convenience stores or rarely leave room/home” on loneliness lost its significance. These results indicate that several demographic and lifestyle factors, including sex, age, marital status, employment status, and outgoing behaviors, were associated with the outcome even after adjusting for Internet use habits as confounding variables.

## Discussions

The significant findings of this study underscore the intricate relationship between demographic, mental health, outgoing behaviors, and internet use patterns with loneliness in the Japanese population. Men, young individuals, singles, and non-working participants faced higher odds of experiencing loneliness. Mental health factors, such as dissatisfaction with life and romance, life stress, and psychological distress, were strong contributors.

The nuanced findings on outgoing behaviors challenge conventional assumptions, revealing that even individuals attending school/work regularly can experience profound loneliness if their outgoing activities are limited. Notably, the impact of outgoing patterns on loneliness is distinct from other mental health factors. Additionally, the impact of internet use habits, while statistically significant, needs careful interpretation due to relatively small effect sizes.

### The influence of demographics on loneliness

The gender dimension stands out, with men being at higher risk of loneliness compared to women. This finding may be indicative of potential differences in social support networks or coping mechanisms between genders. Younger individuals, particularly those in their 10s and 20s, faced significantly higher odds of loneliness, possibly due to life transitions, social pressures, or changes in social relationships common during these age periods, highlighting the vulnerability of young adults to loneliness. Moreover, the marital and romantic status played a crucial role, with singles reporting higher odds of loneliness compared to those who were married, cohabiting, or dating (18). This supports the idea that romantic relationships can serve as protective factors against loneliness. Interestingly, employment status also emerged as a significant predictor, with being employed associated with lower odds of loneliness (18). This could be attributed to the social interactions and support systems that come with a regular job, underscoring the importance of occupational engagement in reducing loneliness.

### The impact of psychological factors on loneliness is evident

Those with unsatisfactory romance and job situations, higher stress levels, and elevated psychological distress (K6 scores >9) were more likely to experience loneliness. This reaffirms the connection between mental well-being and loneliness (21), highlighting that dissatisfaction in both personal and professional spheres can contribute to feelings of social isolation.

### Challenging hikikomori classification

Historically, hikikomori individuals have been categorized based on their outgoing behaviors, with a clear distinction between those who attend school or work regularly and engage in social activities and those who predominantly stay at home. This classification has been a defining criterion for identifying hikikomori individuals. However, our findings suggest that this classification may not accurately capture the experience of loneliness among hikikomori.

This study observed that different patterns of outgoing behavior significantly influenced loneliness scores. Notably, individuals who attended school or work but did not engage in other social activities had the highest mean loneliness scores, indicating a higher level of loneliness. Furthermore, logistic regression analysis revealed that those who reported “going out for work or school but doesn’t go out for other reasons,” “mostly staying at home but occasionally going out for social events,” or “typically staying at home but going to nearby places like convenience stores or rarely leaving their room/home” had significantly higher odds of experiencing loneliness compared to those who were outgoing in multiple aspects of life. These results challenge the conventional understanding of hikikomori solely based on outgoing behaviors. It appears that loneliness can exist even among individuals who attend school or work regularly but do not engage in additional social activities. This prompts us to reconsider whether the current classification of hikikomori adequately represents the loneliness experienced by these individuals.

The intention is not to dismiss existing criteria but to encourage a reassessment of their adequacy in representing the loneliness experienced by individuals with specific outgoing patterns. The study acknowledges the complexity of this relationship and questions the helpfulness of the current classification in fully understanding and addressing the loneliness experienced by those labeled as hikikomori.

### Implications

In-Depth Exploration of Hikikomori Experiences: To address the nuances uncovered, further exploration of the emotional and psychological experiences of individuals classified as hikikomori is crucial. Interventions should prioritize understanding and addressing loneliness within this population.

Nuanced Strategies for Combatting Loneliness: Amid the ongoing challenges of COVID-19, interventions aimed at combating loneliness, especially within the hikikomori context, should adopt nuanced strategies. Recognizing the emotional and psychological intricacies is vital for effective public health initiatives.

### Strengths and limitations

The strength of this study is in the pre-stratification of gender and age and pseudo-randomization during the sample recruitment; therefore, the sample is reasonably representative of the national population in terms of demographic distribution. Second, the sample size was considerably big overall, comprising large samples of male and female participants (N>200), improving the results’ stability. Third, the recruitment was done online through a survey company, which assured the anonymity of participants, reducing chances of reporting bias associated with the participants’ attempt to “look good” or “look smart” in fear of being evaluated. However, online recruitment has limited the generalization of the results to the general population of Internet users. Also, caution should be exercised when applying these findings to diverse populations or considering longitudinal trends as the characteristics and behaviors of Internet users can evolve over time. Sample may represent the people who feel comfortable completing an online study but not those who are not. Finally, the technical limitations in the survey prevented the assessment of the characteristics of non-responders and the dropouts; selection bias was not excluded. The study acknowledges the potential influence of unmeasured confounding factors that were not included in the analysis. Uncontrolled variables could impact the relationship between exposure variables and loneliness, introducing bias or affecting the generalizability of the findings.

## Conclusion

This study has provided valuable insights into the multifaceted nature of loneliness in the Japanese population. It has identified several significant contributors to loneliness, including sex, age, marital status, employment status, and outgoing behaviors. These findings underscore the need for a comprehensive approach to understanding and addressing loneliness, as it is influenced by a complex interplay of demographic and lifestyle factors. The results also shed light on the potential inadequacies of the current classification of hikikomori, as it may not fully capture the loneliness experienced by individuals who attend school or work regularly but do not engage in additional social activities. For future implications, these findings have important implications for policymakers, healthcare providers, and researchers. To combat the loneliness epidemic in Japan, interventions and support mechanisms need to be tailored to the specific needs of different demographic groups. This may include targeted programs for young adults, women, or individuals with specific outgoing patterns. Additionally, our results call for a reevaluation of the hikikomori classification to ensure that individuals’ emotional well-being is adequately addressed within this framework.

Further research is warranted to delve deeper into the emotional experiences of hikikomori individuals and to explore the potential role of the COVID-19 pandemic in exacerbating loneliness. Longitudinal studies could provide valuable insights into the persistence of loneliness and its post-pandemic implications. Moreover, efforts to develop and implement effective interventions to alleviate loneliness in Japan should be a priority. This study opens the door for a more connected and inclusive future, emphasizing the importance of addressing loneliness as a critical public health issue in Japan and beyond.

## Data availability statement

The data analyzed in this study is subject to the following licenses/restrictions: Data Use Agreements: Access to the datasets may require the signing of data use agreements or contracts specifying the terms and conditions for data usage, including restrictions on sharing, redistribution, or publication of the data. Requests to access these datasets should be directed to roselineyong@med.akita-u.ac.jp.

## Author contributions

RY: Conceptualization, Data curation, Formal analysis, Investigation, Methodology, Project administration, Writing – original draft, Writing – review & editing.
