# Corrigendum: Reevaluating hikikomori and challenging loneliness assumptions in Japan: A cross-sectional analysis of a nationwide internet sample

**DOI:** 10.3389/fpsyt.2024.1396070

**Published:** 2024-05-07

**Authors:** Roseline Yong

**Affiliations:** Department of Environmental Health Science and Public Health, Graduate School of Medicine, Akita University, Akita, Japan

**Keywords:** loneliness, hikikomori, social withdrawal, outgoing behaviors, mental health, cross-sectional, secondary analysis, nationwide sample

In the published article, there was an error in the **Title**. Instead of “Reevaluating hikikomori: challenging assumptions and redefining loneliness in Japan”, it should be “Reevaluating hikikomori and challenging loneliness assumptions in Japan: A cross-sectional analysis of a nationwide internet sample”.

In the published article, there was an error in the **Affiliations**. Instead of “Department of Environmental Health Science and Public Health, Akita, Japan”, it should be “Department of Environmental Health Science and Public Health, Graduate School of Medicine, Akita University, Akita, Japan”.

In the published article, there was an error in the **Abstract**, Paragraph 2. This sentence previously stated: “A sample of 623 participants, representative of Japanese internet users aged 16 and above, underwent factor analysis.”

The corrected sentence appears below:

“A sample of 623 participants, representative of Japanese internet users aged 16 and above, was included for analysis.”

In the published article, there was an error in the **Keywords**. Instead of “loneliness, hikikomori, social withdrawal, outgoing behaviors, mental health”, the keywords should be “loneliness, hikikomori, social withdrawal, outgoing behaviors, mental health, cross-sectional, secondary analysis, nationwide sample”.

In the published article, there was an error in **Table 2**, column 4, row 7. The high score for ‘Killing time’, *rarely/never*, was incorrectly captured as “25 (146%)”. The correct score is “25 (14.6%)”.

In the published article, there was an error in **Table 2**, column 3, row 47. The low score for ‘P2P and FTP’, *sometimes*, was incorrectly captured as “42 (11.5%)”. The correct score is “52 (11.5%)”.

In the published article, there was an error in **Table 2**, column 3, row 53. The low score for ‘Online survey or quiz’, *sometimes*, was incorrectly captured as “78 (7.3%)”. The correct score is “78 (17.3%)”.

In the published article, there was an error in **Table 2**, column 3, row 60. The low score for ‘Online shopping/auctions’, *often/always*, was incorrectly captured as “179 (29.6%)”. The correct score is “179 (39.6%)”.

The corrected **Table 2** appears below:

**Table 2:** Distributions of UCLA loneliness scores among different Internet use habits.

		UCLA loneliness scores			Mean	SD	Eta squared
		lower scores	higher scores	*p* value			
Official purpose (study/work)	rarely/never	211 (46.7%)	72 (42.1%)	0.238	43.0	12.1	0.00
	sometimes	81 (17.9%)	26 (15.2%)		41.9	12.0	
	often/always	160 (35.4%)	73 (42.7%)		41.8	12.2	
Stress release	rarely/never	201 (44.5%)	60 (35.1%)	0.001	40.9	12.0	0.02
	sometimes	128 (28.3%)	38 (22.2%)		42.2	10.2	
	often/always	123 (27.2%)	73 (42.7%)		44.4	13.6	
Killing time	rarely/never	105 (23.2%)	25 (14.6%)	0.005	38.9	10.6	0.03
	sometimes	123 (27.2%)	37 (21.6%)		41.8	10.3	
	often/always	224 (49.6%)	109 (63.7%)		44.0	13.2	
Communication	rarely/never	223 (49.3%)	96 (56.1%)	0.267	43.7	12.3	0.02
	sometimes	116 (25.7%)	41 (24%)		42.0	11.7	
	often/always	113 (25%)	34 (19.9%)		39.8	11.8	
Associating	rarely/never	359 (78.8%)	128 (74.9%)	0.026	42.2	12.3	0.00
	sometimes	59 (13.1%)	17 (9.9%)		41.8	11.4	
	often/always	37 (8.2%)	26 (15.2%)		44.4	11.9	
Expanding hobbies and network of friends	rarely/never	341 (75.4%)	132 (77.2%)	0.549	42.6	12.2	0.00
	sometimes	56 (12.4%)	16 (9.4%)		41.3	10.0	
	often/always	55 (12.2%)	23 (13.5%)		41.8	13.7	
Resourcing	rarely/never	26 (5.8%)	5 (2.9%)	0.258	42.1	8.7	0.00
	sometimes	59 (13.1%)	19 (11.1%)		42.6	11.0	
	often/always	367 (81.2%)	147 (86%)		42.4	12.5	
Sharing problems	rarely/never	376 (83.2%)	135 (78.9%)	0.46	42.0	12.2	0.01
	sometimes	50 (11.1%)	23 (13.5%)		44.1	11.1	
	often/always	26 (5.8%)	13 (7.6%)		44.5	12.6	
Building community network	rarely/never	388 (85.8%)	153 (89.5%)	0.421	42.8	12.4	0.01
	sometimes	27 (6%)	9 (5.3%)		40.6	9.5	
	often/always	37 (8.2%)	9 (5.3%)		38.7	10.6	
Online dating	rarely/never	441 (97.6%)	158 (92.4%)	0.011	42.1	12.1	0.01
	sometimes	5 (1.1%)	5 (2.9%)		47.5	10.4	
	often/always	6 (1.3%)	8 (4.7%)		50.6	12.9	
Assessing pornography	rarely/never	371 (82.1%)	121 (70.8%)	0.008	41.5	11.9	0.02
	sometimes	49 (10.8%)	31 (18.1%)		45.4	12.5	
	often/always	32 (7.1%)	19 (11.1%)		45.8	13.0	
Using anonymous online bulletin boards (2ch etc.)	rarely/never	316 (69.9%)	93 (54.4%)	<.001	40.9	11.3	0.03
	sometimes	87 (19.2%)	40 (23.4%)		44.0	12.2	
	often/always	49 (10.8%)	38 (22.2%)		46.9	14.4	
Blogging and SNS	rarely/never	219 (48.5%)	83 (48.5%)	0.894	42.3	12.1	0.00
	sometimes	86 (19%)	35 (20.5%)		43.3	11.4	
	often/always	147 (32.5%)	53 (31%)		41.9	12.7	
Release personal updates and work presentations	rarely/never	380 (84.1%)	139 (81.3%)	0.246	42.4	12.2	0.00
	sometimes	32 (7.1%)	19 (11.1%)		44.2	11.7	
	often/always	40 (8.8%)	13 (7.6%)		40.6	11.8	
Assessing Youtube/iTunes etc.	rarely/never	210 (46.5%)	54 (31.6%)	0.003	40.8	11.0	0.01
	sometimes	114 (25.2%)	53 (31%)		44.1	12.3	
	often/always	128 (28.3%)	64 (37.4%)		43.1	13.3	
P2P and FTP	rarely/never	364 (80.5%)	128 (74.9%)	0.298	42.3	12.4	0.00
	sometimes	52 (11.5%)	25 (14.6%)		42.7	10.3	
	often/always	36 (8%)	18 (10.5%)		42.6	12.7	
Online gaming	rarely/never	382 (84.5%)	134 (78.4%)	0.05	41.9	12.2	0.01
	sometimes	43 (9.5%)	17 (9.9%)		43.5	10.7	
	often/always	27 (6%)	20 (11.7%)		46.0	12.8	
Online survey or quiz	rarely/never	27 (6%)	12 (7%)	0.891	46.0	12.6	0.01
	sometimes	78 (17.3%)	29 (17%)		42.2	11.7	
	often/always	347 (76.8%)	130 (76%)		42.1	12.2	
Financial transaction	rarely/never	227 (50.2%)	96 (56.1%)	0.294	43.2	12.5	0.01
	sometimes	110 (24.3%)	41 (24%)		42.9	11.6	
	often/always	115 (25.4%)	34 (19.9%)		40.0	11.7	
Online shopping/auctions	rarely/never	99 (21.9%)	33 (19.3%)	0.393	41.6	10.8	0.00
	sometimes	174 (38.5%)	60 (35.1%)		42.5	11.6	
	often/always	179 (39.6%)	78 (45.6%)		42.7	13.2	

Chi-square test were used to explore the relationship between loneliness and various everyday internet use habits. To ensure the validity of our statistical tests, the homogeneity of variances using Levene’s and Welch’s tests were assessed. One-way ANOVA to facilitate the comparison of means. Effect sizes, employing Cohen’s classiﬁcation for eta-squared were calculated to gauge the magnitude of effects, small (.01), medium (.06), or large (.14). (N=623).

The author apologizes for these errors and states that this does not change the scientific conclusions of the article in any way. The original article has been updated.

