# Correction: Reevaluating hikikomori and challenging loneliness assumptions in Japan: a cross-sectional analysis of a nationwide internet sample

**DOI:** 10.3389/fpsyt.2026.1834932

**Published:** 2026-06-24

**Authors:** Roseline Yong

**Affiliations:** 1 Department of Mental Health, Graduate School of Medicine, The University of Tokyo, Tokyo, Japan; 2 Department of Environmental Health Science and Public Health, Graduate School of Medicine, Akita University, Akita, Japan

**Keywords:** loneliness, hikikomori, social withdrawal, outgoing behaviors, mental health, cross-sectional, secondary analysis, nationwide sample

The original article was missing one affiliation for author Roseline Yong. The following has been added as a first affiliation: “Department of Mental Health, Graduate School of Medicine, The University of Tokyo, Tokyo, Japan”. The final affiliations now read:

”Roseline Yong^1,2^”

”1 Department of Mental Health, Graduate School of Medicine, The University of Tokyo, Tokyo, Japan

2 Department of Environmental Health Science and Public Health, Graduate School of Medicine, Akita University, Akita, Japan”

The original version of this article has been updated.

